# *In Vitro* Effects of Some Botanicals with Anti-Inflammatory and Antitoxic Activity

**DOI:** 10.1155/2016/5457010

**Published:** 2016-08-15

**Authors:** Gianandrea Guidetti, Alessandro Di Cerbo, Angela Giovazzino, Valentina Rubino, Anna Teresa Palatucci, Sara Centenaro, Elena Fraccaroli, Laura Cortese, Maria Grazia Bonomo, Giuseppina Ruggiero, Sergio Canello, Giuseppe Terrazzano

**Affiliations:** ^1^Division of Research and Development, SANYpet SpA, 35023 Bagnoli di Sopra, Italy; ^2^School of Specialization in Clinical Biochemistry, “G. d'Annunzio” University, 66100 Chieti, Italy; ^3^Department of Translational Medical Sciences, University of Naples Federico II, 80131 Naples, Italy; ^4^Ph.D. School of Science, University of Basilicata, 85100 Potenza, Italy; ^5^Department of Veterinary Medicine and Animal Productions, University of Naples Federico II, 80100 Naples, Italy; ^6^Department of Science, University of Basilicata, 85100 Potenza, Italy

## Abstract

Several extrinsic factors, like drugs and chemicals, can foster autoimmunity. Tetracyclines, in particular oxytetracycline (OTC), appear to correlate with the emergence of immune-mediated diseases. Accumulation of OTC, the elective drug for gastrointestinal and respiratory infectious disease treatment in broiler chickens, was reported in chicken edible tissues and could represent a potential risk for pets and humans that could assume this antibiotic as residue in meat or in meat-derived byproducts. We investigated the* in vitro* anti-inflammatory properties of a pool of thirteen botanicals as a part of a nutraceutical diet, with proven immunomodulatory activity. In addition, we evaluated the effect of such botanicals in contrasting the* in vitro* proinflammatory toxicity of OTC. Our results showed a significant reduction in interferon- (INF-) *γ* production by human and canine lymphocytes in presence of botanicals (^⁎^ *p* < 0.05). Increased INF-*γ* production, dependent on 24-hour OTC-incubation of T lymphocytes, was significantly reduced by the coincubation with* Haematococcus pluvialis*, with* Glycine max*, and with the mix of all botanicals (^⁎^ *p* < 0.05). In conclusion, the use of these botanicals was shown to be able to contrast OTC-toxicity and could represent a new approach for the development of functional foods useful to enhance the standard pharmacological treatment in infections as well as in preventing or reducing the emergence of inflammatory diseases.

## 1. Introduction

 The immune system has the fundamental role of not only protecting and defending the organism against infections but also controlling homeostasis and health maintenance against infections, autoimmune diseases, and tumor onset [1]. Depending on the pathogen or on antigen, two different immune responses can occur: the humoral and the cellular responses [2]. Moreover, the immune system can be classified into two fundamental phases: the innate and acquired (or adaptive) responses [3]. Innate immunity is present in vertebrates and in nonvertebrates, represents the first-line defence in the species and is based on cells (i.e., macrophages, polymorphonuclear cells, and natural killer lymphocytes) and on some mechanisms, mediated by soluble substances (i.e., complement proteins, antibodies, natural compounds, etc.) that defend the plants and animals from infections [4]. Conversely, adaptive immunity is present only in vertebrates and is a host defence related to several specific cellular mechanisms that specifically recognize the antigens and are fundamentally expressed by B and T lymphocytes, plasma cells, and antibodies [5]. The CD4^+^ T helper (T_H_) lymphocytes represent key cells in the polarization of inflammatory/noninflammatory immune response: T_H_1 and T_H_2 are the most common [6]. The T_H_1 response is characterized by the a secretion of INF-*γ*, which optimizes the bactericidal macrophages capability, induces the production of opsonizing and complement-fixing antibodies, and fosters the establishment of an optimal CTL response. The T_H_2 response is characterized by interleukin- (IL-) 4, IL-5, IL-10, and IL-13 release, which results in the activation of B cells to make neutralizing noncytolytic antibodies, leading to humoral immunity [6].

Exacerbation and endurance of T_H_1 response have been associated with the emergence of inflammatory diseases [6] and autoimmune disorders [7]. In particular, INF-*γ* appears to play a pivotal role in inducing autoimmune responses [8–16].

Several extrinsic factors, like drugs and chemicals, can foster the development of autoimmunity [17–22]. In this regard, the use of tetracyclines appears to correlate with the emergence of autoimmune diseases [23–29]. Concerning this, OTC represents the main drug used to control gastrointestinal and respiratory diseases in broiler chickens. Its accumulation was demonstrated in chicken edible tissues [30] and could represent a potential risk also for pets and humans that could assume this antibiotic as a residue in meat or in meat-derived byproducts. Recently, we published two papers evidencing the* in vitro* toxicity of bone meal-derived OTC from intensive poultry farming, in terms of apoptosis induction [31], as well as the proinflammatory cytokines, that is, INF-*γ*, release from peripheral blood mononuclear cells (PBMCs) cultures [32]. Moreover, we evidenced that the presence of significant concentrations of OTC in gym trained human subjects was linked to the presence of food intolerances [33]. Therefore, we hypothesized a possible modulatory activity exerted by a pool of botanicals derived from medical plants, which are successfully used in several commercially available nutraceutical diets. Intriguingly, many botanicals could have the capability to modulate the immune system [34]. In this regard, it is well known that the immunomodulatory activity of acemannan, a mucopolysaccharide extracted from* Aloe vera*, related to modulation of nitric oxide release that modulate classes I and II MHC cell surface antigens involved in antigen presentation [35, 36]. The same immunomodulating activity was observed for fermented* Carica papaya* able to increase T_reg_ cells, reduce INF-*γ* ^+^CD4^+^ T cells, and possibly alter the growth of several cancer cell lines [37–39]. As to Maitake mushroom (*Grifola frondosa*), many reports have shown its ability to downregulate cytokine secretion, such as Tumor Necrosis Factor- (TNF-) *α* and INF-*γ*, as well as to inhibit adhesion molecule expression and cell-mediated immunity enhancement [40–44]. Downregulation of overexpressed cytokines in different inflammatory and immune-related inflammatory conditions was also reported for curcumin extracted from turmeric (*Curcuma longa*) [45–47]. Antiproliferative and chemopreventive effects are known to be also exerted by other curcuminoids, for example, demethoxycurcumin, bisdemethoxycurcumin, and alpha-turmerone [48, 49]. Cytokine downregulation is also performed by* Glycine max* (soybean) isoflavones that interfere with leukocyte endothelial adhesion ability [50–54]. In more detail, isoflavones, that is, genistein, can suppress dendritic cell function and cell-mediated immunity.

It is noteworthy that some botanical principles, which have been investigated in this study such as astaxanthin (from* Haematococcus pluvialis*), resveratrol (from* Polygonum cuspidatum*), and* Cucumis melo*, are characterized by antioxidant and anti-inflammatory properties as well as modulation properties towards CD8^+^ T-cell proliferation [55, 56]. Anti-inflammatory but also oxidative stress preventing activity has been also ascribed to* Cucumis melo* extract due to its high activity on superoxide dismutase [57, 58].

Recently, we published a paper evidencing the role for a nutraceutical diet in regulating the immune response in canine* Leishmaniosis* along with standard pharmacological treatment [59]. In particular, the presence of* Ascophyllum nodosum*,* Cucumis melo*,* Carica papaya*,* Aloe vera*,* Haematococcus pluvialis*,* Curcuma longa*,* Camellia sinensis*,* Punica granatum*,* Piper nigrum*,* Polygonum cuspidatum*,* Echinacea purpurea*,* Grifola frondosa*, and* Glycine max* in the diet correlated with a significant decrease in T_H_1 response, in terms of INF-*γ* production. Such evidence highlighted the anti-inflammatory effects of these specific botanicals. In addition, we suggested the anti-inflammatory effects of several botanicals added to specific diets in relieving inflammatory conditions in chronic pathologies affecting dogs [59–63].

Based on these premises, the aim of our study was to investigate the potential anti-inflammatory properties of those 13 botanicals having immune-modulatory effect as supplemented diet regulating the immune response in* Leishmaniosis* [59]. In particular, we tested the* Ascophyllum nodosum*,* Cucumis melo*,* Carica papaya*,* Aloe vera*,* Haematococcus pluvialis*,* Curcuma longa*,* Camellia sinensis*,* Punica granatum*,* Piper nigrum*,* Polygonum cuspidatum*,* Echinacea purpurea*,* Grifola frondosa*, and* Glycine max* and their ability to counteract the proinflammatory toxicity of OTC* in vitro*.

## 2. Materials and Methods

### 2.1. Culture Medium and Botanicals

To evaluate the cellular production of cytokines, human and canine PBMCs were incubated overnight with an* ad hoc* culture medium. Briefly, the first step was the solubilization of 1 gr of powder of each plant-derived substance in an appropriate chemical vehicle depending on solubility degree. In particular,* Ascophyllum nodosum* (pure powder of* Ascophyllum nodosum* seaweed, laminarin content min. 2.3%, and fucoidans content min. 11.4% [64]),* Aloe vera* (*Aloe vera* gel 200 : 1 powder, aloin content min. 1% [65]),* Cucumis melo* (lyophilized extract of melon, superoxide dismutase min. 1 UI/mg [57]),* Polygonum cuspidatum* (powder obtained from dried* Polygonum cuspidatum* roots, resveratrol content min. 8% [66]),* Camellia sinensis* (standardized decaffeinated green tea leaves powder, catechins content min. 75% [67]),* Carica papaya* (Papaya fermented granular, rich in papain [68]),* Glycine max* (Soy powder, 40% isoflavones [69]), and* Grifola frondosa* (maitake carpophore dry extract, polysaccharides content min. 20.0% [70]) were solubilized in 10 mL of PBS 1x, with the exception of* Glycine max* that was added to 30 mL of PBS 1x to gain the full solubilization.* Haematococcus pluvialis* (standardized beadlets of* Haematococcus pluvialis* extract, astaxanthin content min. 2.5% [71]) was solubilized in 5 mL of dimethyl sulfoxide and 5 mL of PBS 1x.* Echinacea purpurea* (*Echinacea purpurea* dried extract, polyphenols content min 4% [72]),* Piper nigrum* (black pepper powder, piperine content min. 95% [73]),* Curcuma longa* (turmeric dried extract, curcuminoids content min. 95% [74]), and* Punica granatum* (standardized powdered extract from pomegranate, ellagic acid content min. 20% [75]) were solubilized in 4 mL of ethanol and 6 mL of water.

The solubilized botanicals were added to RPMI 1640 culture medium (Sigma-Aldrich, Milan, Italy) to obtain the* ad hoc* medium in the proportion of 1 : 10 (vehicle-solubilized substance : RPMI 1640) to preserve the good quality of cellular condition in the culture.

The cytokine cell production was evaluated in presence of the* ad hoc* medium containing the solubilized individual substance or a mixture containing all the solubilized botanicals. The vehicles employed for the solubilization were used as specific controls in the same proportion of* ad hoc* medium (1 : 10, vehicle : RPMI 1640). The mixture was composed by all* ad hoc* medium from the botanicals in a variable percentage according to that contained in the commercial canine food, previously used as immunomodulating diet able to reduce INF-*γ* production [59]. Briefly, the mixture contained 66.3% of* Ascophyllum nodosum*, 3.1% of* Aloe vera*, 6.1% of* Cucumis melo*, 1.5% of* Polygonum cuspidatum*, 1.5% of* Camellia sinensis*, 3.1% of* Carica papaya*, 4.6% of* Glycine max*, 6.3% of* Grifola frondosa*, 1.1% of* Haematococcus pluvialis*, 3.1% of* Echinacea purpurea*, 0.6% of* Piper nigrum*, 2.3% of* Curcuma longa*, and 1.5% of* Punica granatum*. The obtained mixture was added to RPMI 1640 culture medium to obtain the* ad hoc* medium in the proportion of 1 : 10 (vehicle/mixture : RPMI 1640) to preserve the good quality of cellular condition in the culture.

*Ascophyllum nodosum*,* Aloe vera*,* Cucumis melo*,* Polygonum cuspidatum*,* Camellia sinensis*, and* Haematococcus pluvialis* were purchased from Italfeed S.r.l, Milano (Italy).

*Carica papaya*,* Glycine max*,* Echinacea purpurea*,* Punica granatum*,* Piper nigrum*, and* Curcuma longa* were purchased from Nutraceutica S.r.l, Monterenzio, Bologna (Italy) while* Grifola frondosa* was purchased from A.C.E.F. S.p.a., Fiorenzuola D'Arda, Piacenza (Italy).

All the botanicals are in form of powder and are free from genetically modified organisms (Reg. 1829/2003-1830/2003 EC), gluten, bovine transmissible spongiform encephalopathy, and food allergens (DIR 2003/89/EC and 2006/142/EC).

### 2.2. Human and Canine Donors and Cell Preparation

The human blood collection from 10 healthy donor volunteers (5 males and 5 females, 20–30 years old) was performed at the Haemotrasfusional Center of University of Naples “Federico II,” according to standard procedures and used within the 3 hours from the collection.

Peripheral blood was collected from ten healthy dogs (5 males and 5 females, 5–9 years old and ranging between 15 and 35 kg in weight). All dogs were enrolled with the owner consent in the Department of Veterinary Medicine and Animal Productions, University of Naples “Federico II.” Human or canine PBMCs were isolated by centrifugation on Lymphoprep (Nycomed Pharma) gradients, as previously described [59, 76]. Obtained PBMCs were considered as mixed population of T and non-T lymphocytes.

### 2.3. Monoclonal Antibodies, Detection of Intracellular Cytokine Production, and Flow Cytometry

For the immune-fluorescent staining a panel of fluorescent-labelled monoclonal antibodies (mAbs) was used to evaluate the human CD3, CD8, INF-*γ*, and IL-4, as well as a panel of isotype-matched mAb controls (Becton Dickinson Pharmingen, San Jose, California). In addition, we used several fluorescent-labelled mAbs against canine CD3, CD4, CD8, CD45, INF-*γ*, and IL-4 molecules and isotype-matched controls (Serotec Ltd., London, UK).

To analyze the production of INF-*γ* and IL-4 cytokines, 2 × 10^6^/mL purified PBMCs were incubated overnight (10–12 hours) in the* ad hoc* medium of each botanical or of mixture (see Section 2.1). In particular, to obtain the cytokine production, PBMCs were always cultured in presence of 500 ng/mL of phorbol-12-myristate-13-acetate (PMA) and 1 *μ*g/mL of Ionomycin (Sigma-Aldrich), as described in [77]. To avoid extracellular cytokine export, the cultures were performed in the presence of 5 *μ*g/mL of Brefeldin-A (Sigma-Aldrich), as described in [77].

To test the ability of botanicals in contrasting the toxic role of OTC, we used the commercial preparation of the drug (Oxytetracycline 20%®, TreI, Reggio Emilia, Italy). 1 *μ*g of OTC [31] was added to cell culture and incubated for overnight (10–12 hours) as previously described [32]. In addition,* Haematococcus pluvialis* or* Glycine max* or the mixture* ad hoc* medium was used in the coincubation of cells with OTC and all along the overnight (10–12 hours) culture.

At the end of overnight (10–12 hours) incubation, the above incubated cells were fixed and permeabilized by using a commercial cytokine staining kit following the manufacturer's instructions (Caltag Laboratories, Burlingame, CA, USA). Briefly, the cell fixing and permeabilization procedure were of 20 min at 4°C each. At the end of procedure, PBMCs were washed twice by centrifugation (800 ×g) in RMPI 1640 culture medium.

PBMCs were stimulated overnight with PMA and Ionomycin, cultured in a medium containing the botanicals solubilization buffer (vehicle), and used as control points. The proportion of vehicle and RPMI 1640 was the same of* ad hoc* medium (1 : 10 ratio).

**Figure 1:**
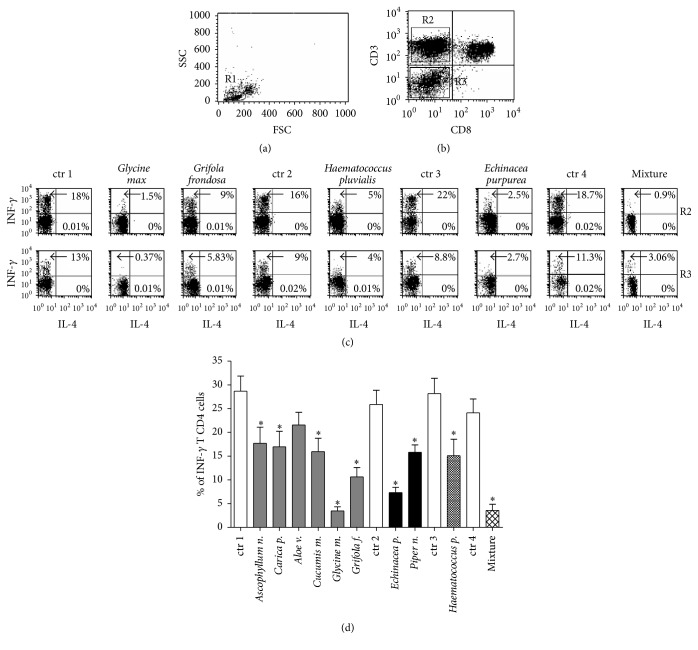
The effects of botanicals on cytokine production by human PBMCs. (a) shows the gating on viable lymphocytes (R1 in dot plot graph) based on FSC and SSC parameters (see Section 2); (b) represents the gating on T_H_ lymphocytes (CD3^+^ CD8^−^ as R2 in the dot plot graph) and on non-T cells (CD3^−^ CD8^−^ cells as R3 in the dot plot graph); and (c) shows the INF-*γ* and IL-4 production in human T_H_ lymphocytes and non-T cells incubated with* ad hoc* medium derived from botanicals or from mixture (see Section 2). Cytokine production was evaluated as percentage of INF-*γ* (*y*-axis) and IL-4 (*x*-axis) producing cells. The percentage of INF-*γ* (upper left quadrant inside the dot plots) and IL-4 (low right quadrant inside the dot plots) producing CD4 T (R2) and non-T (R3) cells are reported. The different cell incubations with* ad hoc* medium derived from botanicals or from mixture (see Section 2) are indicated on the top of each graph. (d) reports the statistic representation of 10 experiments on human CD4^+^ T Lymphocytes evaluated as percentage of INF-*γ* producing cells, ^*∗*^ *p* < 0.05. The different cell-incubations with* ad hoc* medium derived from botanicals or from mixture (see Section 2) are indicated on the top of each column. The abbreviation “ctr” in (c) and (d) indicates the basal cytokine production by PMBCs stimulated by PMA and Ionomycin and in presence of the* ad hoc* medium based on the same solubilizing-vehicle but free from the botanicals (see Section 2); specifically, ctr 1 (*Ascophyllum n.*,* Carica p.*,* Aloe v.*,* Cucumis m.*,* Glycine m.*, and* Grifola f.*), ctr 2 (*Echinacea p.*,* Piper n.*), ctr 3 (*Haematococcus p.*), and ctr 4 (the mixture of all the botanicals).

**Figure 2:**
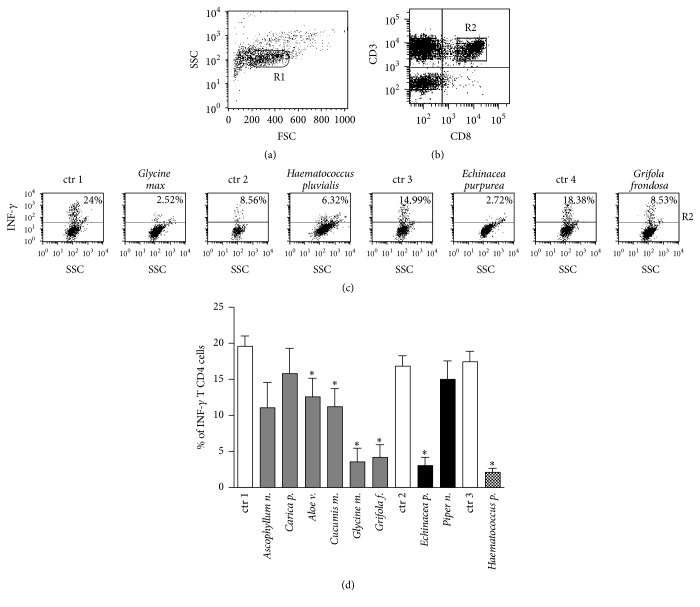
The effects of botanicals on INF-*γ* production by canine PBMCs. (a) shows the gating on viable lymphocytes (R1 in dot plot graph) based on FSC and SSC parameters (see Section 2). (b) represents the gating on CD4^+^ T lymphocytes (CD3^+^ CD8^−^ as R2 in the dot plot graph). (c) reports the results from one representative experiment showing the percentage (the number in upper quadrant) of INF-*γ* producing canine CD4^+^ T lymphocytes gated on R2 (*y*-axis); *x*-axis indicates the SSC parameter (see Section 2). The different coincubations of cells with* ad hoc* medium or mixture (see Section 2) are indicated on the top. (d) shows the statistic representation the INF-*γ* production by canine CD4^+^ T Lymphocytes evaluated as percentage of INF-*γ* producing cells in 10 representative experiments, ^*∗*^ *p* < 0.05. The abbreviation “ctr” in (c) and (d) indicates the basal INF-*γ* production by PMBCs stimulated by PMA and Ionomycin and in presence of the* ad hoc* medium based on the same solubilizing-vehicle but free from the botanicals (see Section 2): specifically, ctr 1 (*Ascophyllum n.*,* Carica p.*,* Aloe v.*,* Cucumis v.*,* Glycine m.*, and* Grifola f.*), ctr 2 (*Echinacea p.*,* Piper n.*), and ctr 3 (*Haematococcus p.*).

The intracellular cytokine production was evaluated by using the triple staining technique and analyzed by flow cytometry (FACSCalibur platform) and CellQuest Software (Becton Dickinson Pharmingen, San Jose, California). The analyzed cells were always gated (R1 in dot plot of Figures 1(a) and 2(a)) on forward scatter (FSC) and side scatter (SSC) FACS parameters (cell size and cell complexity, resp.) to reasonably select the region of viable lymphocytes in order to avoid any interference due to the possible presence of death cells.

### 2.4. Statistical Analysis

Data are presented as the means ± standard error of the mean (SEM) and were firstly checked for normality using the D'Agostino-Pearson normality test. The Kruskal-Wallis followed by Dunn's multiple comparisons analysis was performed. A ^*∗*^ *p* < 0.05 was considered significant. Statistics was performed by GraphPad Prism 6 (GraphPad Software, Inc., La Jolla, CA, USA).

## 3. Results and Discussion

### 3.1. The Anti-Inflammatory Effect of Botanicals as Significant Reduction of INF-*γ* Production in Human T and Non-T Lymphocytes

We focused on INF-*γ* production, as the main proinflammatory cytokine able to foster the T_H_1 and non-T cell immune responses involved in several etiopathogenic mechanisms at the basis of inflammatory-mediated disease [6].

As shown in Figure 1, the overnight incubation (10–12 hours) with each botanical as well as with a mix of all botanicals induced a significant decrease in INF-*γ* production in the T_H_ lymphocytes (CD3^+^ CD8^−^ cells gated as R1 in the dot plot graphs of Figures 1(b) and 1(c) and reported as mean ± SEM of 10 experiments in Figure 1(d)) and in non-T cells, mainly represented by NK lymphocytes (CD3^−^ CD8^−^ cells gated as R2 in the dot plot graphs of Figure 1(c)). In particular, the individual incubation with* Ascophyllum nodosum*,* Cucumis melo*,* Carica papaya*,* Haematococcus pluvialis*,* Curcuma longa*,* Camellia sinensis*,* Punica granatum*,* Piper nigrum*,* Polygonum cuspidatum*,* Echinacea purpurea*,* Grifola frondosa*, and* Glycine max* was able to reduce INF-*γ* production. Intriguingly, despite the obtained slightly decrease in cytokine production, the* Aloe vera* incubation did not induce a significant reduction from the statistical point of view (Figure 1(d)). In this regard, we cannot rule out that a larger number of experiments (more than the 10 performed in this study and summarized in Figure 1(d)) or a higher concentration of substance could confirm the reduction in lymphocyte INF-*γ* production by the incubation with this botanical.

The basal IL-4 production was undetectable or only slightly detectable in T and non-T lymphocytes, as expected in PBMCs from healthy human donors after exposure to PMA and Ionomycin [77] and was not modulated after the overnight incubation with the botanicals (Figure 1(c)). Each specific vehicle, used to solubilize the botanicals, was used as control and the obtained value was substracted from each experimental point to obtain the correction following the formula “the value obtained from cell culture in presence of botanicals – the value obtained from cell culture in presence of the vehicle alone = corrected experimental point value.” It is of note that even if the used vehicles appeared to not induce significant cell death in the culture, the flow cytometry analysis was always performed by gating on viable cells to avoid any possible interference dependent on death cells (see Figure 1(a) and Section 2.3). Moreover, the* ad hoc* medium from botanicals did not exert effect in absence of PMA and Ionomycin stimulation (data not shown).

### 3.2. The Anti-Inflammatory Effect of Botanicals as Significant Reduction of INF-*γ* Production in Canine CD4^+^ T Lymphocytes

The individual incubation with* Ascophyllum nodosum*,* Cucumis melo*,* Aloe vera*,* Haematococcus pluvialis*,* Curcuma longa*,* Camellia sinensis*,* Punica granatum*,* Polygonum cuspidatum*,* Echinacea purpurea*,* Grifola frondosa*, and* Glycine max* was able to significantly decrease the INF-*γ* production in the CD4^+^ lymphocytes (dot plot graphs in Figure 2(c), summarized in Figure 2(d)). In contrast, the incubation with* Carica papaya* or with* Piper nigrum* seemed not to induce a statistically significant reduction (Figure 2(c)). Also, in this case, as referred to in human experiments, we cannot rule out that a larger number of experiments (more than the 10 performed in this study, summarized in Figure 2(d)) or a higher concentration of the substances could confirm the reduction in lymphocyte INF-*γ* production by the incubation with these two botanicals.

IL-4 production was undetectable in T lymphocytes, as expected in PBMCs from healthy dogs after exposure to PMA and Ionomycin [59], and was not modulated after the overnight incubation with the botanicals (data not shown).

The specific vehicles, employed to solubilize the substances, were used as controls and the resulting values were substracted from experimental points, as described (see Section 3.1). Flow cytometry analysis was always performed by gating on viable cells to avoid any possible interference dependent on death cells (see Figure 2(a) and Section 2.3).

### 3.3. The Anti-Inflammatory Effect of Botanicals as Significantly Contrasting Effect on INF-*γ* Production Dependent on OTC Exposure of Human T Lymphocytes

**Figure 3:**
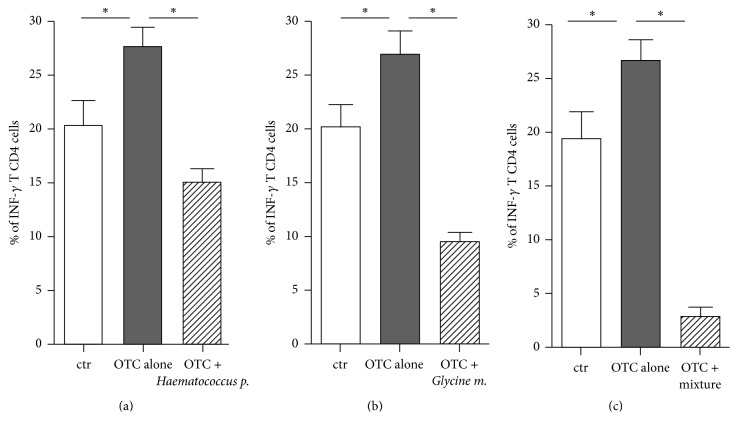
Statistic representation of the INF-*γ* production in human CD4^+^ T Lymphocytes after the OTC exposure and the contrasting effects after botanicals challenge in 10 representative experiments. (a)* Haematococcus p.*; (b)* Glycine m.*; and (c) the mixture of all the botanicals. Cytokine production was evaluated as percentage of INF-*γ* producing T CD4^+^ cells. All the incubations (basal, OTC alone, and OTC + botanical) were performed in the* ad hoc* medium based on the vehicle used to solubilize the botanical, so that the abbreviations “ctr” indicate the basal INF-*γ* production by PMBCs stimulated by PMA and Ionomycin and in presence of the* ad hoc* medium based on the same solubilizing-vehicle but free from the botanicals (see Section 2). ^*∗*^ *p* < 0.05.

Notably, the individual incubation with* Haematococcus pluvialis* or with* Glycine max* was able to contrast the previously demonstrated proinflammatory effect of OTC in human T lymphocytes [32]. Indeed, the increased INF-*γ* production, dependent on 24-hour OTC-incubation of T lymphocytes, was strongly reduced by the coincubation with* Haematococcus pluvialis* or* Glycine max* (Figures 3(a) and 2(b), resp.). Note that the individual incubation with the botanicals, other than* Haematococcus pluvialis* and* Glycine max*, was unable to contrast OTC-toxicity (data not shown), while the mixture of all substances exerted a significant effect. Nevertheless, as referred to in previous sections, we cannot rule out that a larger number of experiments or a higher concentration of each substance could confirm the anti-OTC effect also for the other tested botanicals.

The specific vehicles, used to solubilize the substances, were considered as controls and the resulting values were substracted from experimental points, as described (see Section 3.1).

## 4. Conclusions

This study was inspired by two recently published* in vivo* observations in which we suggested a potential anti-inflammatory effect of some nutraceutical diets, containing the studied botanicals, in infectious and inflammatory diseases [59–61].

In particular, we observed that a diet enriched by* Ascophyllum nodosum*,* Cucumis melo*,* Carica papaya*,* Aloe vera*,* Haematococcus pluvialis*,* Curcuma longa*,* Camellia sinensis*,* Punica granatum*,* Piper nigrum*,* Polygonum cuspidatum*,* Echinacea purpurea*,* Grifola frondosa*, and* Glycine max* was able to reduce proinflammatory T cell responses in canine* Leishmaniosis* [59] and the clinical feature of ear inflammation in chronic otitis in dogs [60].

Here, we observed the* in vitro* effect of* Ascophyllum nodosum*,* Cucumis melo*,* Haematococcus pluvialis*,* Curcuma longa*,* Camellia sinensis*,* Punica granatum*,* Polygonum cuspidatum*,* Echinacea purpurea*,* Grifola frondosa*, and* Glycine max* in reducing* in vitro* proinflammatory cytokine production by human and canine PBMCs. These botanicals appeared to exert a potential anti-inflammatory effect that was evident in the reduction of INF-*γ* production in human T and non-T cells and in canine T lymphocytes. Conversely,* Aloe vera*,* Carica papaya*, and* Piper nigrum* appeared to be ineffective in reducing this cytokine production. These results seem to be contradictory with the data observed in dogs [59], where the diet containing all these botanicals, including* Aloe vera*,* Carica papaya*, and* Piper nigrum*, exerted a therapeutic effect by reducing the inflammatory aspects of* Leishmaniosis*. Such apparent contradiction may be explained by the different sensitivity between* in vitro* and* in vivo*, as well as by the fact that* in vivo* botanicals are probably synergized in the combined administration as in the diet. In this regard, this latter consideration fits with* in vitro* effect obtained by the mixed incubation with all substances that induced the INF-*γ* decrease.

Moreover, as stated in Section 3, we cannot rule out that a larger number of experiments or a higher concentration of substances could confirm the reduction in lymphocyte INF-*γ* production also by* Aloe vera*,* Carica papaya*, and* Piper nigrum*.

Taken together, our observation highlighted the relevance for the use of botanicals to modulate the inflammatory responses in both dogs and humans. Indeed, exacerbation and the persistence of T_H_1 response frequently result in the emergence of inflammatory diseases [6] and autoimmunity disorders [7] and the increase of INF-*γ* production is associated with autoimmunity in humans [8–16]. In addition, some of the botanicals used in this study were previously suggested as antioxidants and immune-modulating substances to reach the physiological status in several models of disease in human [55, 78] and animals [59, 60, 79–83].

Moreover, this study was also inspired by our recent paper, which evidenced the* in vitro* toxicity of OTC in terms of inflammatory response increase by human lymphocytes [32]. In this regard, here we evaluated the potential ability of* Ascophyllum nodosum*,* Cucumis melo*,* Carica papaya*,* Aloe vera*,* Haematococcus pluvialis*,* Curcuma longa*,* Camellia sinensis*,* Punica granatum*,* Piper nigrum*,* Polygonum cuspidatum*,* Echinacea purpurea*,* Grifola frondosa*, and* Glycine max* to contrast the OTC-toxicity exerted* in vitro* in human lymphocytes.

Our data suggested that the incubation with the mixture of these botanicals clearly reduced the OTC-induced INF-*γ* production in T cells. It is of relevance that the individual incubation with* Haematococcus pluvialis* or with* Glycine max* significantly reduced this cytokine production.

Such evidence may shed new light on the misunderstood scenario resulting from the increasing emergence of inflammatory diseases in humans, dogs, and cats [84–89]. Moreover, it has been suggested that tetracycline, in particular OTC, could take part in this scenario and could represent harmful compounds for human health and animals fed meat derived from intensive livestock [25–30, 33, 90].

In conclusion, this study could open an interesting approach regarding the use of anti-inflammatory and antioxidant botanicals in immune-mediated pathologies and in infectious diseases as well as to counteract the effect of several putative toxic substances present in food, such as the OTC, which can cause inflammatory disorders and diseases.
